# Occult Hepatitis B Virus Infection Among Blood Donors in the Capital City of Addis Ababa, Ethiopia: *Implications for Blood Transfusion Safety*

**DOI:** 10.3389/fgstr.2022.887260

**Published:** 2022-07-06

**Authors:** Gizachew Gemechu, Woldearegay Erku Abagez, Dawit Hailu Alemayehu, Abebech Tesfaye, Demewoz Tadesse, Abiy Kinfu, Adane Mihret, Andargachew Mulu

**Affiliations:** ^1^ Department of Microbiology, Immunology and Parasitology, School of Medicine, College of Health Sciences, Addis Ababa University, Addis Ababa, Ethiopia; ^2^ Bacterial and Viral Research Directorate, Armauer Hansen Research Institute (AHRI), Addis Ababa, Ethiopia; ^3^ Medical Department, National Blood Bank, Addis Ababa, Ethiopia

**Keywords:** occult hepatitis B infection, hepatitis B virus, blood donor, chronic hepatitis, hepatocellular carcinoma

## Abstract

**Background:**

Occult hepatitis B virus infection (OBI) remains a potential threat to blood safety in developing countries. Nevertheless, there is no data available on the magnitude of occult hepatitis among blood donors in Ethiopia. Therefore, this study aimed to estimate the magnitude of OBI among blood donors in Ethiopia.

**Objectives:**

The aim of this study is to determine the magnitude of OBI and associated risk factors among blood donors at the National Blood Bank, Addis Ababa, Ethiopia.

**Methods:**

A total of 973 HBsAg-negative plasma samples were tested for anti-HBc antibody using an ELISA and viral DNA using automated ABBOTT real-time PCR. Along with plasma samples, demographic data were retrieved from the database with respect to donors. Both descriptive and inferential statistics were employed for the analysis of data by SPSS 20. *p*-values less than 0.05 were considered as statistically significant.

**Results:**

Of the total of 973 study participants, 445 (45.7%) were female with a mean age of 26.5 years. A total of 144 (14.8%) blood samples were anti-HBc antibody reactive. Four (0.41% of all samples, and 2.8% of anti-HBc-positive samples) samples were confirmed to have OBI by DNA detection. The mean viral load among the confirmed OBI samples was 31 IU/ml with ±12 SD, suggesting true occult hepatitis BV infections. Age was found to be a risk factor for anti-core positivity and was statically significant at *p* = 0.0001.

**Conclusion:**

About four out of 1,000 blood donors screened negative with HBsAg had occult HBV infection. This shows that there could be a risk of HBV transmission through blood transfusion in Ethiopia. Therefore, there is a need for further investigation and action to revise the existing blood screening strategy by including anti-HBc and HBV nucleic acid testing.

## Introduction

Despite the availability of an effective vaccine and potent antiviral treatments, chronic hepatitis B virus (HBV) infection continues to be a major public health issue worldwide (1) and causes liver cirrhosis and hepatocellular carcinoma (HCC) that result in considerable morbidity and mortality (2). According to the World Health Organization (WHO), half of the world population has been infected with HBV (1). With an estimated 257 million people living with chronic infection, the worldwide prevalence of chronic HBV infection in 2016 was reported to be 3.5%. Prevalence was the highest in the African (6.1%) and Western Pacific regions (6.2%) (3). In Ethiopia, meta-analysis results show that the overall prevalence of HBV was 7.4%, and among blood donors, it is higher than an overall population with 8.4% prevalence (4). In fact, HBV has been reported to be a leading transfusion-transmissible viral infection in Ethiopia (5, 6). Data generated by the Ethiopian National Blood Bank from blood screening in 2014 show an average of 3.2% rate of HBV infection among blood donors from different regions of the country (unpublished data).

Occult HBV infection is a state of infection in which surface antigen is undetectable while HBV DNA is in blood, which is undiagnosed frequently (7). Occult hepatitis B infection may be found in blood donors as a result of various clinical conditions, including (1) window period of acute infections (2), end stage of chronic hepatitis B (3), low-level viral replication after recovery from hepatitis, and (4) escape mutants not detected by current HBsAg tests (8, 9).

The global report on occult hepatitis among blood donors varies in different parts of the world, and it ranges from 0.006% (10) to 17.2% (11). Because Ethiopia is also one of the HBV-endemic countries in Africa, occult hepatitis B infection is expected. There is an indicator in a different group of communities: 5.8% and 19.1% among HIV patients in the Eastern and Northern part, respectively (12, 13). A higher rate (20.3%) was also seen among pregnant women from Gondar, Northern Ethiopia (14). Both of the above examples might be showing a glimpse of the actual occult HBV infections in the country, and there is scarcity of data on blood donors. Therefore, the present study was conducted for the first time in Ethiopia to determine the magnitude of occult hepatitis B infection among blood donors in Addis Ababa at the Ethiopian National Blood Bank.

## Materials and Methods

### Study Population

A cross-sectional study was conducted at the National Blood Bank in Addis Ababa. In Ethiopia, as routine blood screening for transfusion, blood banks screen the blood for four transfusion-transmissible diseases: HBV, hepatitis C virus (HCV), human immunodeficiency virus (HIV), and syphilis. They are using Ag/Ab ELISA tests [Wantai Biological Farm (China)] to detect HBsAg, anti-HCV, and HIV 1 + 2, and anti-syphilis Ab ELISA to detect syphilis (Nora Kampitsch, MSc, India).

A total of 973 HBsAg-negative serum samples were collected from December 2020 to April 2021. The minimum sample size was determined according to the probable sampling formula. The samples were picked by simple random sampling from HBsAg screen negative of daily blood collected and brought to National Blood Bank laboratory during the study period.

Leftover plasma samples were aliquoted into tubes (2 ml capacity) and transported to Armauer Hansen Research Institute (AHRI) under a cold chain in the ice box for further tests. Transported samples were stored at −80°C till use. Demographic data such as age, sex, donation history, number of donations, and data regarding the site of collection were retrieved from the National Blood Bank documentation system.

### Sample Processing and Detection of OBI

In this study, all specimens tested negative for HBsAg during the screening procedures at the National Blood Bank. The serum samples collected for the study were retested for further confirmation at AHRI for surface antigen with a commercially available ELISA kit [Monolisa™ HBs Ag ULTRA (France)]. Samples that tested negative with surface antigen were subjected to anti-HBc ELISA test using a commercial kit [Monolisa™ anti-HBc ULTRA (France)]. Samples with a positive result in the anti-HBc ELISA were subjected to HBV viral load quantification in a 200-μl plasma volume using the ABBOTT real-time automated DNA extraction and amplification machine (ABBOTT real-time PCR, Abbott Molecular Inc.). The target sequence for the ABBOTT real-time HBV assay is in the surface gene of the HBV genome. This region is specific for HBV and is highly conserved. The primers are designed to hybridize to this region with the fewest possible mismatches among HBV genotypes A through H (15).

### Data Analysis

All the statistical analyses were performed using SPSS version 20. Both descriptive and inferential statistics were employed for analyzing the data. Frequencies were used to determine the prevalence of occult hepatitis infection among blood donors. Bivariate logistic regression and chi-square test were employed to assess the significantly associated risk factors with occult HBV infection. Prevalence figures were calculated for the total study population, and the association between variables was calculated. *p*-values less than 0.05 were considered statistically significant.

### Ethical Considerations

The project was ethically approved by the Department of Microbiology, Immunology, and Parasitology Research Ethics Review Committee (DRERC), College of Health Sciences, Addis Ababa University (DRERC/01/2021) and exempted from review by the AHRI/ALERT Ethics Committee (protocol number PO/37/20). Blood banks always obtain donors’ written consent, and therefore, we were granted a waiver for this research.

## Results

### Demographic Data

The age of the study participants was between 18 and 61 years, with a median age of 24 years and a mean of 26.5 ± 8.7 years. Of the total study participants, 45.7% were female. There were seven categories of blood collection sites, and the majority (almost 90% of samples) of blood units were collected from schools, blood bank collection centers, and mobile sessions (**Table 1**).

**Table 1:** Sample collection sites and their proportion of samples.

Ser. No	Blood collection sites	Number of samples collected	Percentage (%)
1	Secondary school	270	27.7
2	Blood collection centers	223	22.9
3	Mobile session	335	34.4
4	Colleges	38	3.9
5	Worship place	45	4.6
6	Health facilities	44	4.5
7	Work place (offices)	18	1.8
**Total**		**973**	**100**

### Prevalence of Occult Hepatitis B Virus

**Figure 1:**
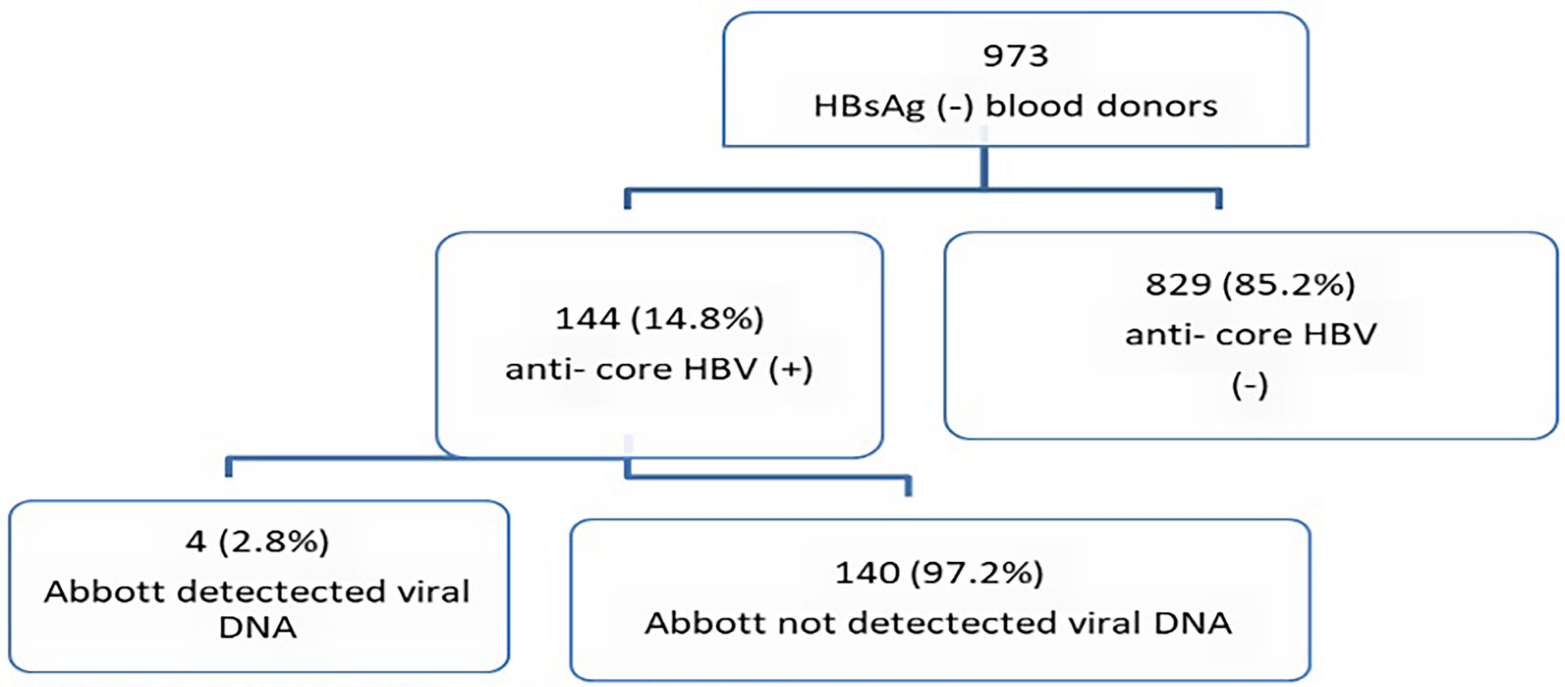
Flowchart of the laboratory process and results.

The seropositivity rate for HBV anti-core among the 973 HBsAg sero-negative blood donors was 144 (14.8%). HBV DNA was detected in 4/144 anti-HBc-positive blood donors, indicating the prevalence of occult HBV infection to be 2.8% among anti-HBc-positive blood donors, and 0.41% among all HBsAg sero-negative blood donors (**Figure 1**). The mean HBV DNA load in viremic HBc^+^ individuals was 31 IU/ml, with a range of 20–48 IU/ml.

### Associated Risk Factors

There was a difference in seropositivity for anti-core between different age groups. It was 42.5% among >45-year-old donors, 27.5% among 36- to 45-year-old donors, and 8% among donors aged between 18 and 25 years. Age was one of the risk factors, which had a statistically significant association (*p* = 0.0001) with anti-HBc seropositivity. On the other hand, increased frequency of blood donation was matched with statistical significance (*p* = 0.001) in the prevalence of anti-core, where 31.8% of blood donors who donated more than eight times had a higher prevalence of anti-HBc compared to first-time donors whose anti-HBc prevalence was only 13.3%. Similarly, age and frequency of previous blood donation were correlated with anti-HBc seropositivity (*p* = 0.0001), where the highest prevalence (42.55%) was observed among the highest age group (46–65 years) compared to the lowest prevalence (7.4%) among the younger age group (18–25 years). The prevalence of anti-core was higher in male participants (16.1%) than in female participants (13.2%), but the difference was not statistically significant (**Table 2**).

**Table 2:** Associated risk factors for Anti-HBc.

Characteristics	Categories	Anti-HBc		Total	Odds ratio	*p*-value
		Anti-HBc negative	Anti-HBc positive*N* (%)			
Age	18–25	510	41 (7.4%)	551		
	26–35	218	55 (20.14%)	273		
	36–45	74	28 (27.45%)	102		
	46–65	27	20 (42.55%)	47		
	Total	829	144	973	0.225	0.0001
Blood donation frequency	0–2	653	102 (13.5%)	755		
	3–5	123	23 (15.7%)	146		
	6–7	23	5 (17.85%)	28		
	>8	30	14 (31.8%)	44		
	Total	829	144	973		0.0001
Sex	F	386	59 (13.25%)	445		
	M	443	85 (16.1%)	528		
	Total	829	144	973		0.239

## Discussion

To reduce the risk of transfusion-transmissible diseases, the WHO recommended mandatory blood screening before transfusion for infectious diseases like HBV for both developing and developed countries. The WHO also plans to reach 100% be hepatitis B virus-free blood transfusion by 2030 (16). However, blood screening in the developing world largely depends on ELISA targeting only HBsAg, which cannot detect occult hepatitis. As a result, there is still a risk of transmission of HBV infections through blood transfusion.

The current study was carried out to find out the level of occult hepatitis occurrence among blood donors, who are thought to play a major role in the transmission of HBV infection to recipients. From the total of 973 HBsAg-negative donated blood samples in Addis Ababa, 144 (14.8%) had serological markers for anti-HBc, which showed a previous exposure or current HBV infection. To our knowledge, there are no recent data on the magnitude of anti-HBc antibody positives among blood donors, and there is only one study that was carried out way back showing a high rate (66%) (17), which cannot reflect the current situation. Compared to the latter report, the anti-HBc positivity rate from our study is significantly lower. Several potential reasons could be attributed to this discrepancy between the two studies. In fact, recent studies on anti-HBc seropositivity in non-blood donor groups also show a lower rate than the 66% reported by Tsega et al. (17), as exemplified by the following reports: 19.5% among children aged 5–8 years from Hawassa city (18), an overall anti-HBc positivity of 21% among HIV-positive adults in three hospitals in eastern Ethiopia (19), and 26.8% among pregnant women from Gondar (14). The possible reason for reduction could be the recent implementation of preventive intervention such as child immunization, pregnant mothers screening for treatment, and blood screening with more sensitive immunoassay starting from 2007 (20).

On the other hand, both higher and lower anti-HBc positivity were reported in domestic and elsewhere globally from blood donors [e.g., higher rates from Brazil (22%) (21), Burkina Faso (20.1%) (22), Pakistan (17.28%) (23), and Cameroon (48.7%) (23, 24), and lower rates from Gondar (6.3%) (25), Mexico (6.4%) (26), and Iran (5.18%) (27)]. One study from Egypt reported 14.2% (10), which is comparable to this finding. Age differences, endemicity of HBV in the general population, and investigation approach might be responsible for such discrepant prevalent reports from the same study groups (healthy blood donors).

Due to the lack of molecular assays for blood screening, there is limited knowledge on the rate of occult hepatitis B infection in developing countries, especially among blood donors in Ethiopia. The present study showed a 4/144 (2.8%) prevalence of confirmed occult hepatitis among anti-HBc-positive blood donors. This result was lower than the ones reported from other findings, where it was 5.6% among HIV-negative patients, 6% among HIV patients, and 19.1% among pregnant women (13, 14). This discrepancy is expected as all the latter study participants were members of risk groups. Regarding HIV negative individuals, the endemicity of HBV in the respective local areas and age group differences could be possible reasons. The median age of the participants was 24 years in our study, which is much less than the median age of 40 years indicated by Ayana et al. (13). On the contrary, there was a comparable prevalence rate documented in some countries [e.g., Pakistan (2.9%) (23) and Brazil (2.7%) (21)], and a lower rate from few African countries [e.g., 0.56% from Cameroon (24), 0.1% from Libya (28), and 0.025% from South Africa (29)]; the 2.8% prevalence rate among blood donors[110] in this study is significantly lower than the rates reported from many countries including those from Sudan (16%) (30), Egypt (17.2%) (10), Nigeria (17%) (31), Burkina Faso (20.1%) (22), and Saudi Arabia (8.6%) (32).

The 4/144 HBV DNA positivity we found may not mean that only blood units were OBI positive, as there are three scenarios where OBI may be missed by this study: those seronegative individuals who are negative for both anti-HBc and anti-HBs, which can comprise 1%–20% of all OBI cases (33); in the case of OBI with anti-HBs detectable, while negative for anti-HBc (31); and those anti-HBc seropositive individuals with undetectable HBV DNA because of the low level of cccDNA replication under OBI conditions (34). This implies that the magnitude of occult hepatitis could be more than what we found; thus, it should be a concern.

In regard to HBV viral load, the maximum viral load detected in occult hepatitis B infection in this study was 48 IU/ml, which is higher than those reported from Nigeria (1.6 IU/ml) (31), Cameroon (<6 IU/ml; median, 5 IU/ml) (24), and China (14 IU/ml) (35), but lower than those reported from Saudi Arabia (186 IU/ml) (32), Italy (108 IU/ml) (36), and Lao PDR (3,510 IU/ml) (37). This study shows that age and frequency of donation were found to be associated risk factors for anti-HBc detection, and the findings from the report from Cameroon and Turkey support the argument (24, 38). The possible reason for aged donors having a higher seropositivity for anti-HBc than younger ones could be the fact that as age increases, the exposure rate to HBV will increase. In the case of Ethiopia, older people were born before the introduction of different health system programs to reduce community exposure to the disease.

## Conclusions and Recommendations

In conclusion, this study has shown that almost every 15 of 100 HBsAg-negative blood units collected during the study period that were considered to be “safe and ready-for transfusion” were obtained from individuals who had previous exposure to HBV. Among those blood units that came from individuals who had a history of HBV exposure, 2.8% were confirmed to have occult hepatitis, which has the potential to infect blood recipients, that means there is a possibility of four blood units in every 1000 considered to be safe in the current algorithm of blood screening in Ethiopia will be transfused with HBV unknowingly. If we roughly estimate the rate of OBI among 300,000 blood units donated last year in Ethiopia, we end up with 1,230 potential infectious blood units supposed to be transfused to recipients, which maintains HBV circulation in the community through blood transfusion. Hence, HBsAg screening alone is not sufficient to eliminate the risk of HBV transfusion transmission. Thus, further investigation should be undertaken to get a full picture of the magnitude of occult hepatitis in blood donors. Accordingly, revising the blood screening strategy by including anti-HBc and HBV nucleic acid testing when possible is warranted.

## Data Availability Statement

The original contributions presented in the study are included in the article/supplementary material. Further inquiries can be directed to the corresponding author.

## Ethics Statement

The project was reviewed and approved by the Department of Microbiology, Immunology & Parasitology Research Ethics Review Committee (DRERC), College of Health Sciences, Addis Ababa University (DRERC/01/2021) and exempted from review by AHRI/ALERT Ethics committee (protocol number PO/37/20). Blood Banks always obtain donors written consent and therefore we were granted with waiver for this research

## Author Contributions

GG designed the proposal, collected data and samples, did laboratory work, analyzed data, and wrote the manuscript. WA, AdM, and AnM designed the proposal and reviewed the manuscript. DHA, AT, DT, and AK were involved in laboratory work and sample collection. All authors contributed to the article and approved the submitted version.

## Conflict of Interest

The authors declare that the research was conducted in the absence of any commercial or financial relationships that could be construed as a potential conflict of interest.

## Publisher’s Note

All claims expressed in this article are solely those of the authors and do not necessarily represent those of their affiliated organizations, or those of the publisher, the editors and the reviewers. Any product that may be evaluated in this article, or claim that may be made by its manufacturer, is not guaranteed or endorsed by the publisher.
